# The effect of chronotype on social functioning in schizophrenic patients: examining the chain-mediating role of sleep quality and anxiety

**DOI:** 10.3389/fpsyt.2025.1574414

**Published:** 2025-06-27

**Authors:** Zheng Luo, Jing Zhang, Maoting Guo, Dongmei Wu

**Affiliations:** ^1^ School of Nursing, Chengdu Medical College, Chengdu, Sichuan, China; ^2^ Department of Nursing, Sichuan Provincial People's Hospital, School of Medicine, University of Electronic Science and Technology of China, Chengdu, China

**Keywords:** schizophrenia, chronotype, sleep quality, anxiety, social functioning, chain mediation

## Abstract

**Introduction:**

This study examines the relationship between chronotype and social functioning in individuals with schizophrenia, specifically assessing the mediating roles of sleep quality and anxiety symptoms.

**Methods:**

785 Chinese patients with schizophrenia (aged 18-60) completed assessments using the Morningness–Eveningness Questionnaire (chronotype), Pittsburgh Sleep Quality Index (sleep quality), General Anxiety Disorder-7 (anxiety), and Personal Social Performance Scale (social functioning). Hayes’ SPSS process macros were used for analysis.

**Results:**

Significant associations were found, with chronotype directly impacting social functioning. Indirect mediation effects occurred through two pathways: anxiety alone, and a chain involving sleep quality and anxiety.

**Discussion:**

These findings contribute to understanding how chronotype influences social functioning in schizophrenia, offering insights for recovery support.

## Introduction

Social functioning—the ability to fulfill societal roles through employment, family engagement, community participation, and relationships (1)—is a critical recovery indicator and quality-of-life predictor in severe mental disorders (2, 3). Schizophrenia disrupts cognitive, emotional, and behavioral capacities (4), driving social withdrawal and isolation that reinforce mental health stigma.

Research consistently links social functioning to lifespan, physical health, and psychosocial resilience in individuals with severe mental disorders (5, 6), suggesting that enhancing social function is the focus of mental health management. Nevertheless, its psychological and behavioral mechanisms remain poorly characterized (7, 8). This study systematically examines key determinants and pathways underlying social functioning in schizophrenia, with dual objectives: advancing neurodevelopmental frameworks and guiding preventive clinical strategies.

### From chronotype to social functioning

Chronotype is a pivotal aspect of circadian rhythm, encompassing individual preferences and habits in daily activity patterns and sleep-wake cycles, which vary based on genetic, environmental, and age-gender factors (9). Chronotypes categorize individuals as morning, intermediate, or evening types (10). The association between schizophrenia and chronotype is crucial, potentially impacting both the disorder’s course and its pathophysiology (11). Previous studies suggest that chronotype is a stable trait influenced by endogenous factors and can affect biological and psychological functions (12).

Current research predominantly focuses on young adults and adolescents in investigating the association between chronotype preferences and social functioning. Recent studies indicate that adolescents with stronger nocturnal chronotypes are more likely to report impaired emotional self-regulation (13), reduced daytime functioning (14), and difficulties in demonstrating prosocial behaviors (15). These findings suggest a plausible link between late sleep preferences, compromised social-emotional cognition, and social deficits.

Although research indicates a link between chronotype and social functioning in diverse populations, no studies specifically explore this relationship within schizophrenia, marking a significant gap. Therefore, based on this gap in knowledge, we propose the hypothesis 1: chronotype directly and significantly influences social functioning in schizophrenic patients.

### Sleep quality as a mediator

The association between chronotype and sleep quality has been thoroughly documented across various studies. Merikanto et al. found that individuals characterized as evening-types frequently experience inadequate sleep, nightmares, and a higher prevalence of recent hypnotic use compared to other chronotypes (16). Other studies have shown that i individuals with evening-type reported more pathological and insomnia-related symptoms than morning and intermediate chronotypes (17), as well as a predisposition to morning drowsiness and sleep deprivation (16). Additionally, in a study involving people with schizophrenia, patients with evening-types exhibited poorer sleep quality than those with intermediate or morning chronotypes (18).

Recent research elucidates a portion of the connection between sleep quality and social functioning. Studies focusing on occupational performance indicate that greater sleep dysfunction correlates with increased absenteeism, workplace accidents, and impediments in career advancement within community samples (19). This association has consistently been observed across diverse populations. For instance, an experimental study involving 18 healthy adults aged 18–24 years demonstrated that sleep deprivation led to social withdrawal and feelings of loneliness (20). Studies in older adults from Taiwan and the United States have similarly found that higher levels of social isolation coincide with greater sleep disturbances and poorer sleep quality (21). Moreover, poor sleep has been linked to impaired decision-making and reduced social functioning (22). In schizophrenia, up to 80% of patients experience sleep disturbances that significantly impact their functioning (23).

Based on the aforementioned findings, we propose hypothesis 2: Sleep quality acts as a mediating factor in the relationship between chronotype and social functioning in schizophrenia.

### Anxiety as a mediator

One potential factor influencing the relationship between chronotype and social functioning is anxiety. Multiple studies have robustly established a connection between chronotype and anxiety: chronotype has consistently been linked with anxiety in youth (24–26). Evening types appear to exhibit higher vulnerability to anxiety (27), and individuals with anxiety disorders show a greater preference for evening chronotypes compared to those without such diagnoses (28). Conversely, morning preference has been acknowledged as a protective factor against psychiatric disorders (29). Individuals with morning chronotype tend to adopt healthier lifestyles, including regular exercise and dietary habits (30), which are associated with improved psychological well-being and reduced mental health challenges (31). These factors may partly elucidate the relationship between chronotype and anxiety symptoms.

Furthermore, several studies have identified notable correlations between dysregulated affect and diminished social functioning in patients diagnosed with affective and non-affective psychotic disorders (32–34). Specifically, studies on bipolar disorder indicate that elevated levels of anxiety significantly contribute to reduced social functioning, underscoring the significance of achieving symptom remission (35, 36). Extending these findings, similar trends have been observed among patients with schizophrenia, where heightened anxiety correlates with poorer social functioning (37). At the severe end of the psychosis spectrum, anxiety is hypothesized to mediate the relationship between impairment in social functioning and psychosis (38, 39). This hypothesis is supported by prior research demonstrating that anxiety exacerbates functional impairment in psychosis, thereby worsening social functioning (38).

Drawing from the aforementioned evidence, we posit Hypothesis 3: Anxiety symptoms serve as a mediator in the relationship between chronotype and social functioning.

### The chain mediating role of sleep quality and anxiety

The literature analysis indicates that sleep quality and anxiety symptoms are crucial in mediating the link between chronotype and social functioning. Specifically, how does sleep quality impact anxiety symptoms in individuals with schizophrenia? Evidence shows a significant positive correlation between the Pittsburgh Sleep Quality Index (PSQI) score and the Hamilton Anxiety Rating Scale (HAMA) score (40). Individuals with higher anxiety levels report poorer sleep quality and shorter nocturnal sleep duration than the general population. Diminished sleep quality and duration have been associated with elevated anxiety levels the subsequent day. Henry found that schizophrenic patients exhibit heightened levels of alexithymia (41), and those with higher levels of alexithymia show a stronger association between poorer sleep quality and increased next-day anxiety (42).

This study formulated a chain mediation model to examine whether chronotype influences social functioning through its effects on sleep quality and anxiety symptoms. Although previous research indicates an association between chronotype and social functioning, the specific psychological and behavioral mechanisms remain unexplored. Building on prior studies, this research aims to elucidate the direct and mediated roles of sleep quality and anxiety symptoms in the relationship between chronotype and social functioning. We propose Hypothesis 4: Chronotype exerts a significant indirect influence on social functioning via its effects on both sleep quality and anxiety symptoms.

## Materials and methods

### Participants

This cross-sectional study, conducted in October 2020, aimed to assess the chronotype and social functioning of community-dwelling individuals diagnosed with schizophrenia. Participants were selected from a community in Chengdu, Sichuan Province, based on inclusion criteria that included age (18–60 years), diagnosis by two attending psychiatrists according to the Diagnostic and Statistical Manual of Mental Disorders, and individuals with comorbid conditions (e.g., substance use disorders, neurological illnesses) were excluded to minimize confounding variables that could independently affect sleep quality, anxiety, or social functioning.

A total of 900 questionnaires were distributed, with 818 recovered (recovery rate: 90.89%). After excluding 33 unqualified responses, 785 schizophrenic patients (52.74% males and 47.26% females) aged 23 to 59 years participated in the study (efficacy rate: 95.97%).

Approval for the study was granted by the Medical Ethics Committee of Chengdu Fourth People’s Hospital, and all participants provided informed consent prior to their involvement in the survey.

### Measure

#### Chronotype

The Morning and Evening Questionnaire-5 (MEQ-5) served as a well-validated self-report tool to assess chronotype (43). Comprising five items, the MEQ-5 evaluated participants’ sleep habits. Based on total scores ranging from 4 to 25 points, participants were categorized into evening-types (4 to 11 points), neutral-types (12 to 17 points), and morning-types (18 to 25 points). In a recent study of Chinese psychiatric patients, the MEQ-5 demonstrated strong reliability and alignment with objective sleep measures, supporting its use here (44). Cronbach’s alpha of the scale in this study was 0.706.

#### Sleep quality

The Pittsburgh Sleep Quality Index (PSQI) version utilized in this study is a 19-item retrospective self-report questionnaire covering the preceding 7 days. The assessment covers seven domains, including subjective sleep quality, sleep latency, duration, habitual efficiency, disturbances, medication use, and daytime dysfunction. Scores range from 0 to 3 per component, yielding a total score from 0 to 21, where higher values signify worse sleep quality (45). Cronbach’s alpha of the scale in this study was 0.717.

#### Anxiety

The Generalized Anxiety Scale (GAD-7) with 7 symptom items was developed by Spitzer et al. (46). And has been widely used in scientific research and clinical practice because of its high reliability and validity, simplicity and ease of operation. It has seven questions with ordinal ratings ranging from 0 to 3 for each one, for a maximum of 21, and the total score of 0~4 points was not without anxiety; 5 to 9 are classified as mild anxiety; 10~14 is classified as moderate anxiety; ≥15 is classified as severe anxiety. In 2010, the GAD‐7 was translated into Chinese by He (47) and demonstrated robust reliability and validity among participants from China (48). Cronbach’s alpha of the scale in this study was 0.960.

#### Personal social performance

The Personal and Social Performance (PSP) scale uses a 100-point single-item rating system to evaluate functioning across four core domains: Self-care, social activities, relationships, and behaviors associated with disturbance and aggression. Ratings are assigned based on a continuum from severely impaired to excellent functioning, encompassing all four domains within a scale range of 0 to 100. The reliability of the PSP has been extensively evaluated, demonstrating strong internal consistency (49). Cronbach’s alpha of the scale in this study was 0.844.

### Statistics

In this study, IBM SPSS 26 facilitated comprehensive data analysis. Initially, descriptive analysis outlined the demographic characteristics of participants. Due to the non-normal distribution of the data, Spearman correlation analysis explored associations among chronotype, sleep quality, and social functioning. Subsequently, the PROCESS program (Model 6), developed by Hayes (50), was employed to examine potential serial multiple mediation effects involving sleep quality and anxiety in the relationship between chronotype and social functioning. The mediation analysis examined three indirect pathways: Indirect 1: Chronotype → Sleep quality → Social functioning; Indirect 2: Chronotype → Anxiety → Social functioning; Indirect 3: Chronotype → Sleep quality → Anxiety → Social functioning. A significance level of p < 0.05 was used for all analyses, with bootstrap confidence intervals (CI) set at 95% and 5,000 bootstrap samples. A mediating effect was considered significant if the 95% CI did not encompass zero.

## Results

### Common method deviation test

The Harman single-factor method was employed as a statistical control measure to mitigate potential methodological biases (51). Statistical analysis revealed that the eigenvalue of the first factor accounted for 30.54% of the variance, which was below the critical threshold of 40%. Additionally, the cumulative variance explained by three factors with eigenvalues greater than 1 indicated that common method variance did not significantly influence the outcomes of this study.

### Descriptive characteristics of the sample and correlation analyses

**Table 1** presents the demographic profile of the 785 individuals included in the study. The median age of participants was 34 years, with an interquartile range (25th percentile [P25] = 21years, 75th percentile [P75] = 40 years). A majority of the participants were male (52.74%). The distribution of residence indicated that over 80% of the patients resided in rural areas, with unequal proportions living in towns (11.00%) and cities (3.80%). The majority of schizophrenic patients are currently married (47.60%), with a significant portion who have never been married (32.90%), and others who have experienced divorce (16.20%) or the loss of a spouse (2.30%). Regarding employment status, the majority of schizophrenic patients are unemployed (70.70%), while a portion are employed, with 7.10% holding full-time jobs, 4.50% working part-time, and only 0.30% being retained in their positions. Additionally, some have retired (2.20%), and some are students (0.30%). In terms of financial status, most patients consider it to be average (56.10%), while some perceive themselves as relatively poor (29.40%) or very poor (10.80%), and a small minority consider their financial situation to be good (3.60%) or very good (0.10%). The majority of schizophrenic patients do not currently engage in cigarettes or alcohol consumption, accounting for 72.50% and 93.10% respectively.

**Table 1:** Demographic characteristics of study participants (N=785).

Variables	Category	N	Percentage (%) or Median (P25, P75)
Age		785	34 (21,40)
Gender	Male	414	52.74
	Female	371	47.26
Current residence	Rural	669	85.20
	Town	86	11.00
	City	30	3.80
Marital status	Never married	258	32.90
	Married	374	47.60
	Divorced	127	16.20
	Widowed	18	2.30
	Else	8	1.00
Employment situation	Students	2	0.30
	Unemployed	555	70.70
	Part-time	35	4.50
	Full-time	56	7.10
	Remain at post	2	0.30
	Retired	17	2.20
	Else	118	15.00
Financial situation	Very poor	85	10.80
	Relatively poor	231	29.40
	Ordinary	440	56.10
	Relatively good	28	3.60
	Very good	1	0.10
Smoking	No	569	72.50
	Yes	205	26.10
	Smoking cessation	11	1.40
Drinking	No	731	93.10
	Yes	54	6.90

SD, Standard Deviation.

Analysis of variance (ANOVA) revealed significant differences across chronotype groups in sleep quality, anxiety symptoms, and social functioning (**Table 2**). Evening-type participants exhibited the highest Pittsburgh Sleep Quality Index (PSQI) scores (M = 4.95, SD = 4.11), indicating poorer sleep quality, followed by neutral-types (M = 4.29, SD = 3.02) and morning-types (M = 3.71, SD = 2.73), F = 4.86, p = 0.008. Anxiety symptoms, measured by the Generalized Anxiety Disorder-7 (GAD-7), were most severe in evening-types (M = 3.95, SD = 4.99), moderate in neutral-types (M = 3.41, SD = 3.79), and lowest in morning-types (*M* = 2.09, *SD* = 3.22), F = 14.35, p < 0.001. Social functioning, assessed via the Personal and Social Performance (PSP) scale, followed an inverse pattern: morning-types demonstrated the highest functioning (M = 75.36, SD = 14.43), followed by neutral-types (M = 69.57, SD = 17.24) and evening-types (M = 64.00, SD = 21.34), F = 15.48, p < 0.001. These results underscore systematic variations in key outcomes across chronotypes, with evening-types consistently exhibiting the least favorable profiles.

**Table 2:** Comparison of sleep quality, anxiety symptoms, and social functioning across chronotypes with ANOVA results.

Chronotype	N (%)	PSQI Score (Mean ± SD)	GAD-7 Score (Mean ± SD)	PSP Score (Mean ± SD)
Evening	40 (5.1)	4.95 ± 4.11	3.95 ± 4.99	64.00 ± 21.34
Neutral	322 (41.0)	4.29 ± 3.02	3.41 ± 3.79	69.57 ± 17.24
Morning	423 (53.9)	3.71 ± 2.73	2.09 ± 3.22	75.36 ± 14.43
ANOVA Results		F=4.859 P=0.008	F=14.351 P<0.001	F=15.482 P<0.001

As shown in **Table 3**, spearman’s correlations revealed significant associations: an evening-oriented chronotype correlated with poorer sleep quality (r = -0.137, p < 0.01) and higher anxiety symptoms (r = -0.245, p < 0.01), while a morning-oriented chronotype was linked to better social functioning (r = 0.235, p < 0.01); poorer sleep (r = -0.132, p < 0.01) and elevated anxiety (r = -0.325, p < 0.01) both demonstrated negative relationships with social functioning, with anxiety exhibiting the strongest adverse effect.

**Table 3:** Descriptive statistics of the sample and correlations of all variables.

Variables	Chronotype	Sleep quality	Anxiety	Social functioning
Chronotype	1.000			
Sleep quality	-0.137**	1.000		
Anxiety	-0.245**	-0.264**	1.000	
Social functioning	0.235**	-0.132**	-0.325**	1.000

**p<0.01 (two-tailed).

### Mediation effect test

The mediation analysis revealed that chronotype exerted a significant negative effect on sleep quality (β = -0.2058, p < 0.0001). When considering chronotype and sleep quality as predictors, both variables significantly influenced anxiety levels (β = -0.2310, p < 0.0001 and β = 0.2281, p < 0.001, respectively). In a model incorporating chronotype, sleep quality, and anxiety as predictors, chronotype positively predicted social functioning (β = 0.1487, p < 0.001), while anxiety symptoms negatively predicted social functioning (β = -0.2099, p < 0.001). Notably, sleep quality did not demonstrate a significant effect on social functioning (**Table 4**).

**Table 4:** Regression analysis between variables.

Regression equation		Global fit index			Significance of regression coefficient	
Outcome variable	Predictor variable	R	R^2^	F	β	t
Sleep quality	Chronotype	0.2194	0.0481	6.5585	-0.2058	-4.9039***
Anxiety	Chronotype	0.3532	0.1248	15.8213	-0.2310	-4.5874***
	Sleep quality				0.2281	5.3851***
Social functioning	Chronotype	0.3723	0.1386	15.6091	0.1487	4.3025***
	Sleep quality				-0.0111	0.3196
	Anxiety				-0.2099	-5.8952***

Adjusted age, gender, marriage, number of psychiatric medications, employment situation and financial situation.

***p< 0.001 (two-tailed).

**Figure 1:**
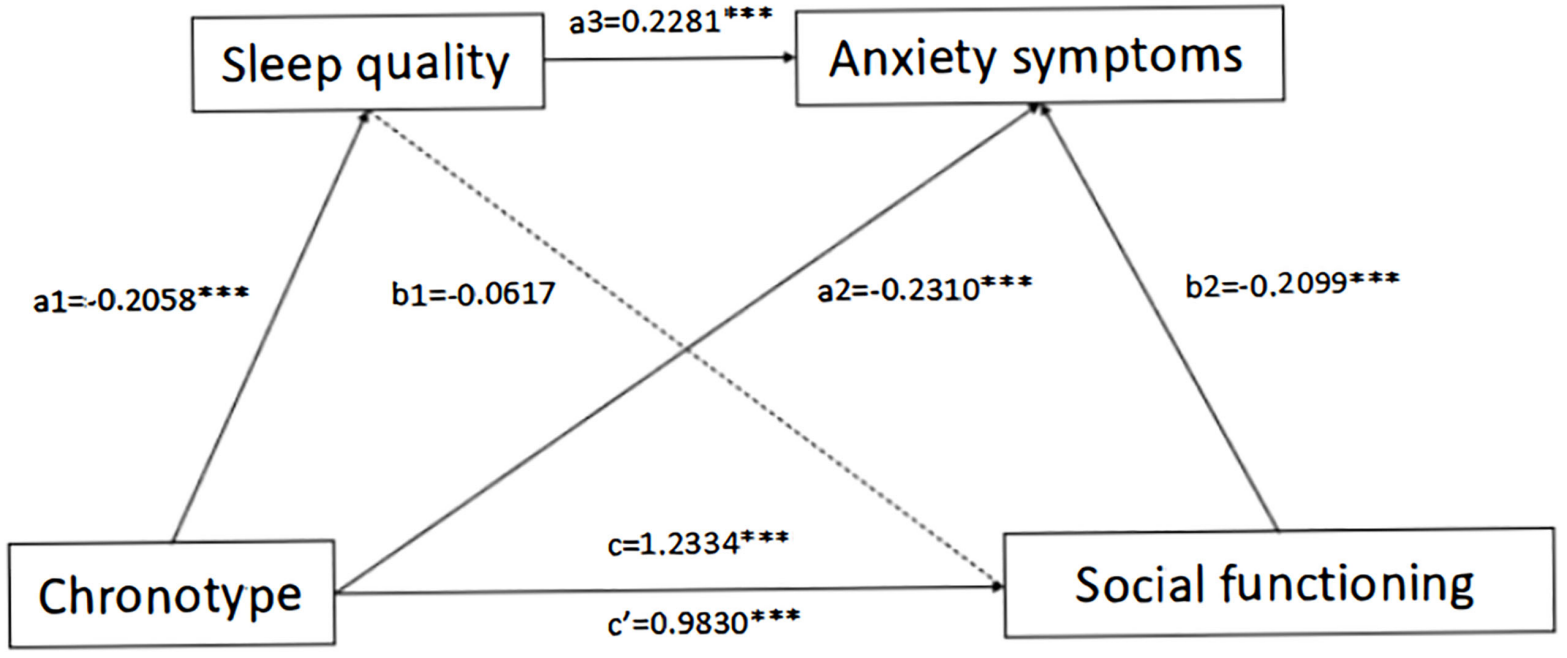
The chain mediation model of schizophrenic patients’ sleep quality and anxiety symptoms.

The serial mediation of sleep quality and anxiety on the relationship between chronotype and social functioning are depicted in **Figure 1** and **Table 5**. The total and direct effects of chronotype on social functioning were significant, with effect sizes of 1.1647 and 0.8931, indicated that patients who preferred evening chronotype were prone to social dysfunction, and patients with evening chronotype could affect social function by affecting sleep quality and anxiety symptoms.

**Table 5:** Hypothesized serial mediation model of sleep quality and anxiety between chronotype and social functioning (n=785).

Model Pathway	Effect	SE	Boot LLCI	Boot ULCI
Total effect (c)	1.2334	0.2267	0.7883	1.6785
Direct effect (c’)	0.9830	0.2285	0.5345	1.4315
Total indirect effect	0.2505	0.0886	0.0866	0.4363
Indirect1	-0.0127	0.0445	-0.1012	0.0784
Indirect2	0.2187	0.0792	0.0819	0.3876
Indirect3	0.0445	0.0165	0.074	0.0815

Indirect 1, Chronotype→ Sleep quality→ Social functioning; Indirect 2, Chronotype→ Anxiety → Social functioning; Indirect 3, Chronotype→ Sleep quality →Anxiety→ Social functioning. BootLLCI, bootstrapping lower limit confidence interval; BootULCI, bootstrapping upper limit confidence interval; SE, standard error; Effect, non-standardized regression coefficient.

In addition, while Indirect 1 did not reach significance, both Indirect 2 and Indirect 3 pathways along with the direct path demonstrated statistical significance. Particularly, Indirect 2 indicated that anxiety symptoms significantly mediated the relationship between chronotype and social functioning, with a coefficient of 0.2187. The Indirect 3 pathway revealed a significant mediation of the influence of chronotype on social functioning by both sleep quality and anxiety symptoms, with an effect magnitude of 0.0445. In conclusion, these findings imply that anxiety symptoms exert a partial mediating function, whereas sleep quality acts as a complete mediator in the relationship linking chronotype to social functioning.

## Discussion

This study investigated predictors of social functioning in schizophrenia by analyzing the interplay of chronotype, sleep quality, and anxiety. While chronotype exhibited a direct effect on social functioning (supporting Hypothesis 1), the hypothesized direct link between sleep quality and social functioning was absent. This unexpected finding aligns with the concept of a “suppressing effect”, where indirect pathways (e.g., chronotype → sleep quality → anxiety → social functioning) may persist despite nonsignificant direct associations (52, 53). For instance, evening chronotypes were linked to poorer sleep quality, which in turn heightened anxiety, ultimately impairing social functioning. Thus, Hypothesis 3 (anxiety as a mediator) and Hypothesis 4 (chain mediation via sleep quality and anxiety) were validated, whereas Hypothesis 2 (sole mediation via sleep quality) was unsupported. These results emphasize the need to consider anxiety as a critical intermediary and highlight the complex, nonlinear pathways through which chronotype impacts social outcomes.

### Theoretical implications

#### The direct effect of chronotype on social functioning

This study constitutes the initial investigation into the link between chronotype and social functioning in schizophrenia. Hypothesis 1 has been substantiated: there exists a positive correlation between chronotype and social functioning within this cohort. A potential explanation lies in circadian rhythm disruptions, particularly a preference for later chronotypes, which may interact with behavioral and neurobiological dysfunctions such as impaired cognition and mood dysregulation. These factors collectively contribute to diminished social functioning (54). Importantly, our findings align with Adan et al. (55), who reported that schizophrenia patients exhibit objective circadian disruptions (e.g., delayed phase, reduced amplitude in distal skin temperature) even when self-reported chronotypes appear intermediate. For instance, patients with intermediate chronotypes in their study still showed impaired daytime activation, suggesting that self-reported preferences may mask underlying biological dysfunction. This underscores the need to explore both subjective chronotype and objective circadian markers (e.g., temperature rhythms) in future studies to fully elucidate their roles in social functioning. Consequently, while chronotype remains a practical clinical indicator, greater attention should be given to integrating multidimensional circadian assessments to mitigate functional decline in schizophrenia.

#### The mediating role of anxiety

Our hypothesis 3 is supported by evidence that anxiety symptoms mediate the relationship between chronotype and social functioning. Specifically, individuals with an evening chronotype tend to experience higher levels of anxiety, contributing to poorer social functioning. Previous studies have consistently shown an association between chronotype and anxiety (56), suggesting that chronotype may influence anxiety processes. Research indicates that anxiety is more prevalent among individuals with evening chronotypes compared to those with morning types or healthy individuals (57, 58). The underlying mechanism may involve circadian disruption affecting melatonin and glucocorticoid secretion, both implicated in mental health issues (50, 59). Additionally, circadian rhythms and clock genes impact neurotransmitter levels, such as dopamine, serotonin, and norepinephrine, which are linked to mood regulation (60). Higher levels of anxiety correlate with greater impairments in social functioning in schizophrenia, consistent with previous findings (37). Anxiety exacerbates functional impairments in psychosis, leading to reduced social functioning and lower quality of life (38). While psychosis contributes to social dysfunction through paranoia and social withdrawal, anxiety independently impairs social functioning by reinforcing negative self-beliefs and discouraging social interactions (46). This cycle of social withdrawal leads to negative relationship experiences and increased avoidance of social situations (39). Thus, anxiety symptoms not only heighten threat anticipation but also perpetuates a cycle of social avoidance in schizophrenia, exacerbating social functioning impairments.

#### The serial multiple mediation model

The mediation analysis revealed two significant pathways: First, individuals with an evening chronotype exhibited heightened anxiety symptoms, which subsequently mediated poorer social functioning; Second, evening chronotype was associated with reduced sleep quality, which in turn exacerbated anxiety symptoms, ultimately contributing to diminished social functioning. Sleep quality alone did not directly affect social functioning. Building on prior findings, our study established that chronotype positively predicted sleep quality (13), which subsequently influenced anxiety levels (61), thereby contributing to diminished social functioning (62). Specifically, individuals with an evening chronotype, often referred to as night owls, are more susceptible to experiencing nightmares (18), encountering difficulties in initiating sleep, and suffering from prolonged sleep latency (32). These factors collectively contribute to lower sleep quality, exacerbating negative mood states among patients and ultimately resulting in a decline in their social functioning. This may be explained by differences in homeostatic sleep regulation among people with different chronotypes, as evening types experience slower dissipation of sleep pressure (63), leading to poorer sleep quality. Moreover, poor sleep quality not only exacerbates psychotic symptoms in schizophrenic patients (62) but also increases the prevalence of physical problems, further deteriorating their overall condition and contributing to anxiety symptoms (64). Finally, elevated levels of anxiety can exacerbate dysfunction in schizophrenic patients (38), leading to negative self-evaluation, social withdrawal, and ultimately reduced social functioning (39, 46).

#### Practical implications

The results of this study may represent a significant step toward implementing interventions to enhance social functioning in individuals with schizophrenia. Given the potential roles of sleep quality and anxiety symptoms in mediating the relationship between chronotype and social functioning, this evidence can guide strategies to improve social outcomes by addressing sleep quality and reducing anxiety symptoms among individuals with schizophrenia. It is important to note that labeling nocturnal individuals negatively is not the intention; however, existing literature suggests that evening-type individuals may be more susceptible to entering vicious cycles. Addressing this issue fundamentally requires raising awareness among evening-type individuals about the risks associated with their chronotype and promoting healthy sleep habits. Psychiatric staff should also prioritize the mental health of individuals with evening chronotypes in schizophrenia, implementing interventions aimed at effectively enhancing their social functioning.

#### Summary of study strengths and limitations

While this study has advanced our comprehension of the relevancy between chronotype and social functioning in schizophrenic patients, several limitations must be acknowledged.

Strengths:

Innovative Research Focus: The study explores a novel area of mental health by linking chronotype, sleep quality, anxiety, and social functioning in schizophrenia.

Large Sample Size: The sample of 785 patients provides robust statistical power, enhancing the reliability and applicability of the findings.

Limitations:

Cross-Sectional Design: This design limits the ability to draw causal conclusions about the relationships between variables.

Sample Homogeneity: The study’s findings may not be generalizable beyond the single community in Chengdu, which affects their broader applicability to diverse populations.

Methodological Rigor: The study employs validated measures and a suitable statistical approach, enhancing the trustworthiness of the results. However, addressing confounding factors such as the influence of specific demographic characteristics on the outcomes would strengthen this section of the methodology. Firstly, this study did not collect data on antipsychotic medications or symptom severity (e.g., positive/negative symptoms), which may influence sleep quality, anxiety, and social functioning. Secondly, although sex was adjusted for in analyses, differential pathways between males and females were not explicitly tested. Finally, while our exclusion of comorbid substance use disorders aimed to isolate the effects of chronotype, sleep, and anxiety, this may limit the generalizability of our findings, as dual disorders are prevalent in schizophrenia and can independently influence social functioning. In the future, these factors should be taken into consideration to obtain more scientific and rigorous results.

Measurement Tool Specificity: While the MEQ-5 has now been validated in a Chinese mental disorder cohort, its specificity for schizophrenia requires further investigation. Future studies should compare the MEQ-5 and rMEQ in schizophrenia populations to determine optimal tools. Moreover, our reliance on self-reported chronotype (MEQ-5) limits our ability to assess biological circadian disruptions such as phase delay or reduced amplitude. Adan et al. (55) demonstrated that schizophrenia patients exhibit significant circadian dysfunction in distal skin temperature, even when self-reported chronotypes appear normal. Future studies should integrate objective measures (e.g., DST, actigraphy) to quantify circadian phase and amplitude, providing a more comprehensive understanding of their role in social functioning.

Results Validity: The results show statistically significant findings, but attention must be paid to non-significant pathways to clarify the interpretations properly. Furthermore, discussing the clinical implications of the findings would make the results more actionable.

Data Interpretation: While the interpretation aligns with the hypotheses, offering deeper insights into unexpected findings (e.g., non-significant links) will provide a more nuanced understanding of the study’s impact.

## Conclusions

This study highlights anxiety as a critical mediator between chronotype and social functioning in schizophrenia, with sleep quality contributing indirectly via anxiety. The results underscore the necessity of prompt action for schizophrenic patients suffering from anxiety symptoms, particularly those with an evening chronotype and poor sleep quality. Hence, medical facilities should identify potential factors contributing to social dysfunction in schizophrenia and intervene promptly to support these individuals. Given the limitations of cross-sectional designs, future longitudinal studies should concurrently investigate sex-specific interactions between chronotype, sleep quality, and anxiety while examining the long-term effects of chronotype-aligned interventions (e.g., light therapy) on social functioning in schizophrenia, controlling for medication effects and symptom severity, to inform optimized personalized intervention strategies. Finally, while our study highlights the role of chronotype in social functioning, circadian disruptions in schizophrenia are multifaceted. Combining self-reported chronotype with objective measures like distal skin temperature could provide a more holistic understanding of how circadian rhythms influence functional outcomes. Such an approach may also identify novel targets for chronobiological interventions to improve social engagement.

## Data Availability

The original contributions presented in the study are included in the article/Supplementary Material. Further inquiries can be directed to the corresponding author.
